# Case Report: An exploration of the neurodevelopmental phenotype of five patients with 48,XXYY during early childhood years

**DOI:** 10.3389/fendo.2025.1688851

**Published:** 2025-12-18

**Authors:** Margaret Olaya, Carole Samango-Sprouse, Debra Counts, Antonie D. Kline, Francie Mitchell, Elizabeth Buscema, Elizabeth Tipton, Teresa Sadeghin, Andrea L. Gropman

**Affiliations:** 1 Department of Research, The Focus Foundation, Davidsonville, MD, United States; 2 Department of Pediatrics, George Washington University, Washington, D.C., United States; 3 Florida International University, Department of Human and Molecular Genetics, Miami, FL, United States; 4 Department of Pediatrics, Sinai Hospital, Baltimore, MD, United States; 5 Harvey Institute for Human Genetics, Greater Baltimore Medical Center, Baltimore, MD, United States; 6 Department of Pediatric Medicine, St. Jude Children’s Research Hospital, Memphis, TN, United States

**Keywords:** 48,XXYY, sex chromosome aneuploidy, X and Y chromosomal disorder, neurodevelopment, early hormone therapy

## Abstract

**Background:**

48,XXYY is a sex chromosome aneuploidy (SCA) occurring in 1:18,000–50,000 male births, characterized by androgen deficiency in conjunction with hypogonadism, hypertelorism, clinodactyly, pes planus, radioulnar synostosis, increased height velocity, hypotonia, and a suspected increased incidence of autism spectrum disorder (ASD). The neurodevelopmental phenotype includes motor dysfunction, speech/language disturbance, and intellectual deficits.

**Aim:**

This series will compare the neurodevelopmental profile of five patients with 48,XXYY during early childhood.

**Methods:**

Five cases of male patients with 48,XXYY were followed beginning at the time of diagnosis. Each case underwent a combination of neurodevelopmental, oral motor, speech/language, physical therapy, medical genetics, and/or neurology evaluations.

**Results:**

In the five cases presented, there was an increased incidence of torticollis, with the right side more common. Abnormal muscle tonus was noted in all cases, characterized by hypotonia of the trunk, upper extremities, and oral motor musculature. Four of the patients exhibited an increased head circumference (≥ 79th percentile) by 7 months of age. All cases had speech/language and motor delays evident in the first 12 months of life and showed no signs of ASD prior to 3 years of age.

**Conclusions:**

The presentation of 48,XXYY is varied, including oral motor deficits, hypotonia, positional and congenital muscular torticollis, respiratory issues, and inner-ear dysfunction. Early presentations of infantile developmental dyspraxia are evident by 18 months, specifically as discrepancies between fine and gross motor and expressive and receptive language skills. This series provides additional insight into the phenotypic presentation of male patients with 48,XXYY during infancy and early childhood and identifies common complications.

## Introduction

48,XXYY is a sex chromosome aneuploidy (SCA) that occurs in approximately one in 18,000–50,000 live male births (1–3). While there is a paucity of literature on very young male subjects with 48,XXYY, the preliminary phenotypic presentation in infancy has been described retrospectively in few studies (3–6). Physical manifestations may include increased height velocity, truncal hypotonia, hypertelorism, clinodactyly, pes planus, and radioulnar synostosis (3, 5). These patients typically experience androgen deficiency in association with hypogonadism, which is treated with hormone replacement therapy (HRT) during adolescence (4–6). Tremors, tics, and seizures have been reported in some patients with 48,XXYY, but the natural history of these manifestations is not well understood (1, 5–8). The neurodevelopmental phenotype presents with speech and gross motor dysfunction, significant language-based learning disabilities, and potentially depressed intellectual abilities (3, 5). Common behavioral manifestations include mood instability, poor social functioning, and a suspected increased incidence of autism spectrum disorder (ASD) (7, 9–14).

Developmental milestones of infants with 48,XXYY have rarely been reported in the literature, with one study reporting the average age for walking to be around 18 months and first words to be around 24 months (2). Neurocognitively, patients with 48,XXYY exhibit a verbal IQ that is typically depressed in comparison to their performance IQ (2, 4, 5, 7, 15). This is consistent with the common presentation of male patients with X and Y chromosomal variations and is typically more complex due to the multiple additive Xs and Ys. Men with 48,XXYY have presented with language-based reading disorders that have affected various aspects of reading, including reading comprehension (3–5, 10, 15). Gross motor deficits are typically present within the first year of life and continue to manifest as they grow, particularly in poor coordination and motor planning skills (3, 5, 16). These neurocognitive challenges often give rise to subsequent difficulties in the academic setting, which may be related to some, but not all, behavioral challenges (11, 17).

Behaviorally, an increased incidence of attention-deficit hyperactivity disorder (ADHD) has been widely reported in adolescents with 48,XXYY (5, 10, 12). Both inattentive and hyperactive subtypes have been noted and are often accompanied by distractibility, poor organizational skills, and impulsivity associated with executive dysfunction (7, 10). Anxiety and social challenges have also been described in 48,XXYY (17, 18). These difficulties are associated with language-based learning disorders with language formulation dysfunction, therefore leading to increasing social frustration (5, 17, 18).

An increased incidence of ASD has also been documented, with one study revealing that children with 48,XXYY may be 20 times more likely to receive a diagnosis of ASD than the general population (14). There have also been several reports of increased aggression, irritability, and rule-breaking behavior in 48,XXYY, progressing with age (12, 17, 19). Srinivasan et al. (12) found that more adolescents with 48,XXYY met criteria for oppositional defiant disorder (ODD) than neurotypical children. However, this study and several others may be skewed by small sample sizes and the failure to account for other influencing factors such as family history, HRT status, and the presence of generalized anxiety disorder and ADHD.

In recent literature, the impact of HRT on patients with an additive X has shown positive treatment effects on neurocognitive abilities, speech and language development, neuromotor capabilities, and behavioral challenges (5, 6, 12). Early hormonal treatment (EHT) has been found to be especially beneficial for neurodevelopmental capabilities in infants and toddlers with 47,XXY, 48,XXXY, and 49,XXXXY (20–24). EHT may be administered during the “minipuberty” stage of an infant’s development, which is between 1 and 6 months of age, because testosterone is known to be associated with brain development, masculinization, and social skills (25–27). The EHT treatment consists of three intramuscular injections, one per month, of 25 mg of testosterone enanthate between 4 and 12 months of age. EHT, ideally administered at 4, 5, and 6 months respectively, has been associated with an increase in expressive and receptive language skills, gross motor abilities, and attentional capacities during the childhood years in male patients with additional Xs (21, 23).

To our knowledge, there has been no documentation of the effects of EHT in boys with 48,XXYY in the research literature. Reports of testosterone therapy in this population have most often been described in adolescence or adulthood (28, 29). Tartaglia et al. (2) reported that 14 of 22 male patients with 48,XXYY were receiving testosterone treatment, though the average age for initiation of administration was 14.9 years with a range of 11–31 years. The introduction of HRT during adolescence has been found to decrease aggressive behavior, suggesting a stabilizing effect of testosterone on behavioral dysfunction during puberty (2, 10, 28, 29). Although these studies indicate positive treatment effects on behavioral outcomes for 48,XXYY, none report the effects of HRT on neurodevelopmental capabilities.

The neurodevelopmental phenotype of patients with 48,XXYY during infancy has not been reported in the research literature, as the average age of detection for this disorder is approximately 7 years of age. During early development, parents reported global developmental delays in speech and motor domains.

This prospective case series describes the medical and neurodevelopmental phenotypes from infancy to early childhood for five patients diagnosed with 48,XXYY through comprehensive evaluations. Each child was assessed by a trained multidisciplinary team that cares for a large cohort of children with X and Y chromosomal variations. All evaluations took place over a period of 3 years, and each evaluation was conducted by the same clinicians from each specialty to ensure continuity. The cases for this report represent all patients with a confirmed 48,XXYY diagnosis who received at least two evaluations from our team.

## Clinical report

### Case 1

#### Diagnosis

Case 1 was prenatally detected as at risk for 47,XYY via noninvasive prenatal screening (NIPS), and his mother chose to forego amniocentesis confirmation. 48,XXYY was confirmed after birth by cord blood with chromosomal microarray (CMA). He received four neurodevelopmental evaluations at 3, 9, 12, 21, and 29 months; a medical genetics evaluation at 2 and 21 months; a pediatric physical therapy evaluation at 12 and 21 months; and a pediatric oral motor as well as speech and language evaluation at 21 months.

#### Birth history

Case 1 was born to a 31-year-old, G5P3Mis2, white woman. Conception was completed through intrauterine insemination, and fetal movement was felt between 16 and 18 weeks of gestation. The mother reports severe stress during the pregnancy, resulting in weight loss, secondary to NIPT results. No other pregnancy problems were reported. He was born with a head-first vaginal presentation at 39 weeks, weighed 3.1 kg (6.8 lbs, 30th percentile), and measured 49.5 cm (19.5 in, 42nd percentile) in length at birth.

#### Perinatal history

Mother and infant stayed in the hospital for two nights. The infant had no difficulties with latching to the breast for feeding, though he did have a lip tie. He alternated between breastfeeding and bottle-feeding throughout the first year of life. He received EHT at 4, 5, and 6 months of age.

#### Medical genetics/pediatric neurology evaluation— 2 and 21 months

At 2 months of age, he weighed 4.6 kg (10 lbs, 3 oz, 15th percentile), had a length of 21.3 cm (8.4 in, fourth percentile), and a head circumference of 40 cm (15.7 in, 88th percentile). He showed no dysmorphic features and no hypertelorism. He presented with a normocephalic head, normal distribution of hair, and a normal forehead. His ears were typically formed and in standard position. His nose was also normally formed with an unremarkable nasal bridge and philtrum. His mouth was typical with an intact palate, no teeth, and a normal chin. His hands were normally formed with no clinodactyly, unremarkable elbows with normal creases bilaterally, and typically formed nails. He showed no birthmarks and was active and alert. Reflexes were not evaluated because the examination was completed through telemedicine during the coronavirus disease 2019 (COVID-19) pandemic.

At 21 months of age, this patient presented with some clumsiness, awkwardness in movement, and has remained hypotonic. He showed poor coordination and body awareness. Balance reactions were inconsistent, such as random falling with little provocation, such as slight shifts in movement or small obstacles in his way. He was evaluated by a pediatric pulmonologist because of chronic coughing and nasal congestion, and tracheomalacia was confirmed. No other pulmonary issues were identified.

#### Physical therapy evaluation— 12 and 21 months

At 12 months, this patient utilized crawling as the major form of mobility; the overall pattern showed good speed and alignment, with a minor amount of pelvic wobble and a tendency to use shoulder internal rotation to increase speed. The pull-to-stand pattern was immature; he primarily used his shoulders with minimal activity of the lower extremities, particularly on the left. Once cued, he could use the half-kneel transition on the right. In standing, he was reluctant to accept weight on the full foot, preferring to use the toes. Cruising is emerging, mainly to the right; however, he did not cruise between surfaces unless he was cued. The mother reported that he is crawling up stairs but not yet going down. Sitting patterns showed a preference for a static circle sit. With slight cueing, he showed increased pelvic mobility in his transitions in and out of sitting. He was unable to stand independently.

Overall, the patient showed mild low tone and joint hyperextensibility. Good antigravity cervical and thoracic extension was seen in sitting and crawling. He also showed good upper shoulder and thoracic extension during supported wheelbarrow play. Normal range of motion was found in the hips and ankles, with webbing between the third and fourth toes. Elbows had mild hyperextensibility with dimpling. Good upper body strength was also noted by his ability to hold a larger ball with his arms while sitting without balance issues. There is mild hip instability due to weakness in the hip extensors and abductors.

At 21 months, the patient’s mother reported that he had been walking for the past 3–4 months and is not using orthotics. During the session, he showed a good variety of movement transitions in all antigravity positions. The most recent gross motor skill was stepping up and down small steps, 1- and 2-in high. He stepped over objects well and showed good control of squat to stand. He had some difficulty with backward movement over the ball, which may indicate some vestibular issues.

Normal range was noted throughout the trunk and extremities. The only asymmetry was a mild right head tilt when walking. Mild hyperextensibility in the wrists, hands, and ankles was still observed. Heel eversion was within norms for his age. The gait pattern showed a mild asymmetry with a tendency to internally rotate on the left, indicating that the right leg is stronger at this time.

The Bayley Scales of Infant and Toddler Development, Fourth Edition (BSID-IV) was completed at both 12 and 21 months for this patient and indicates standardized motor scores, as shown in **Table 1**.

**Table 1:** Case 1 neurodevelopmental testing.

Bayley Scales of Infant and Toddler Development, 4th Edition (BSID-IV)						
Subtest		3 months	9 months	12 months	21 months	29 months
Cognitive	Scaled Score	11	6	10	10	7
	Composite Score	105	80	100	100	85
	**Composite Percentile Rank**	**63**	**9**	**50**	**50**	**16**
Language	Receptive Language Scaled Score	9	10	7	4	5
	Expressive Language Scaled Score	10	8	8	2	2
	Composite Score	98	95	86	63	66
	**Composite Percentile Rank**	**45**	**37**	**18**	**1**	**1**
Motor	Fine Motor Scaled Score	12	10	8	9	8
	Gross Motor Scaled Score	11	7	5	9	7
	Composite Score	109	90	79	95	84
	**Composite Percentile Rank**	**73**	**27**	**8**	**37**	**14**
Preschool Language Scale - 5 (PLS-5)						
Subtest		3 months	9 months	12 months	21 months	29 months
Auditory Comprehension	Standard Score	108	119	96	80	92
	**Percentile Rank**	**70**	**90**	**39**	**10**	**30**
Expressive Communication	Standard Score	114	100	93	85	82
	**Percentile Rank**	**82**	**50**	**32**	**16**	**12**
Early Language Milestone Scale - 2 (ELM-2)						
Subtest		3 months	9 months	12 months	21 months	29 months
Expressive Language	Standard Score	110	75	81	75	69
	**Percentile Rank**	**75**	**5**	**10**	**5**	**2**
Receptive Language	Standard Score	119	119	81	92	81
	**Percentile Rank**	**90**	**90**	**10**	**30**	**10**

Values indicated in bold are percentile rank values based on the standard scoring manuals for each test.

#### Pediatric oral motor/speech and language evaluation— 21 months

In communication, this patient interacted well with his family and the therapist, and his play skills were within normal limits. At the time of the evaluation, he was minimally verbal with some functional single words. There was a functional gap between receptive and expressive skills. He demonstrated inconsistency and atypicality in his articulation and expressive language skills. Observations of the orofacial complex showed decreased tone of the labial, lingual, and buccal musculature. Limited dissociation of movement in the orofacial complex was noted overall. He demonstrated lip seal; however, his typical oral rest posture was open with low-lingual posture. Nasal breathing and lip closure were expected for his age, but given the low muscle tone, it would not be uncommon to see an open oral resting posture.

An intraoral exam revealed excessively thick tissue at the maxillary labial frenum. When assessing the range of motion in his upper lip, it could not be extended fully. Blanching was noted with minimal extension. His mother confirmed that she was told that he had a lip tie when he was a newborn, but no intervention was recommended at that time. His latch to the breast was shallow, but it improved as he grew, and the size of his mouth helped accommodate the tension in his lip and its inability to turn out while latched. Although attachment could not be viewed fully, tension and a limited range of motion were observed. Functional range of motion was observed during feeding.

At 14 months of age, he was recommended to have a swallow study due to frequent nasal congestion and coughing. Tracheomalacia was identified as the cause of his swallowing difficulties. His soft palate was not functioning appropriately, and nasal reflux was causing his chronic congestion. Thickened liquids were recommended at that time. The swallow study was repeated at 21 months, and he was cleared for thin liquids as improvement was seen. His diet varied in taste and texture. He generally took appropriately sized bites but occasionally overstuffed his mouth secondary to decreased muscle tonus in oral facial musculature.

During the feeding evaluation, this patient used his back molars in the absence of lingual lateralization to transport the food from the tongue tip. He had a combination of sucking/softening and munch chewing. A rotary chewing pattern was not observed, which would have been age-appropriate. He chewed with his mouth open, at times using his fingers to readjust food. Pocketing in his cheeks was noted, which is common with weakness in the buccal muscle. He successfully drank regular milk from a thin straw cup without choking or throat clearing.

The tethered oral tissues appeared to severely restrict the range of motion in his upper lip and tongue. There was a clear functional impact of the lack of movement on feeding and speech development. This has prevented the lingual, labial, and buccal muscles from achieving the movement needed to develop strength, coordination, and dissociation from the jaw. These actions are necessary to complete the required movements for articulation and feeding. He will be at a higher risk for picky eating and a self-restrictive diet over time, secondary to this primitive pattern.

#### Neurodevelopmental evaluation—3, 9, 12, 21, and 29 months

Case 1 received four evaluations within the first 2 years of life at 3, 9, 12, and 21 months. The following neurocognitive assessments were completed during each evaluation: the BSID-IV, the Preschool Language Scale, Fifth Edition (PLS-5), and the Early Language Milestone Scale, Second Edition (ELM-2) (**Table 1**).

At 3 months, he presented with speech and language skills within normal limits on the ELM-2, PLS-5, and BSID-IV. He had decreased muscle tone in the orofacial musculature secondary to associated truncal hypotonia. He also exhibited hypotonia in the upper and lower extremities. He had developed right-sided torticollis, but his mother reports it was improving. Both fine and gross motor scores on the BSID-III were within normal limits, with a cumulative motor score in the 73rd percentile. All primitive neonatal reflexes were present. He presented with age-appropriate cognitive skills, exhibiting good state organization and control with appropriate socialization skills. His head circumference was 41.2 cm (16.2 in) at the 73rd percentile. He was 18.9 cm (23.4 in) in length at the 19th percentile and weighed 5.9 kg (13 lbs) at the 27th percentile.

Case 1 received EHT prior to his 9-month evaluation. At 9 months of age, a discrepancy emerged between his receptive language skills and his expressive language skills. On the PLS-5, he scored above average in receptive language (at the 90th percentile) and average in expressive language (at the 50th percentile), showing an obvious discrepancy. On the ELM-2, this discrepancy was even wider; he scored in the 90th percentile for receptive language skills and in the fifth percentile for expressive language skills. The expressive scale of the ELM-2 requires the child to produce specific sounds and words at critical ages, which is helpful in identifying speech delays at these young ages.

On the BSID-IV, he exhibited age-appropriate fine motor skills, but depressed gross motor skills with decreased muscle tone in the core, upper extremities, and lower extremities. This is an early presentation of infantile developmental dyspraxia (IDD) affecting speech and gross motor skills, which typically evolves into childhood apraxia of speech and developmental dyspraxia. His head circumference was 46 cm (18.1 in) at the 79th percentile. He was 70.5 cm (27.8 in) in length at the 27th percentile and weighed 9.9 kg (21.8 lbs) at the 84th percentile.

At 12 months of age, he exhibited age-appropriate receptive and expressive language skills on the PLS-5. He understood a variety of words and requests, and began utilizing gestures and vowel sounds communicatively. However, his gross motor abilities remained depressed in comparison to neurotypical peers. Cognitive and social skills revealed age-appropriate skills with no signs of ASD. His head circumference was 47.5 cm (18.7 in) at the 82nd percentile. He was 76.2 cm (30.0 in) in length at the 41st percentile and weighed 11.5 kg (25.4 lbs) at the 93rd percentile.

At 21 months of age, he showed age-appropriate cognitive skills on the BSID-IV, with a score in the 50th percentile. However, his speech and language scores remained depressed (at the first percentile), with the expressive language score still lower than the receptive language score. This pattern continued on the ELM-1, with expressive and receptive language scores in the fifth and 30th percentiles, respectively. Expressive language skills were consistent with the at-risk status of IDD, as described by Samango-Sprouse and Rogol (30). His motor abilities were within normal limits but slightly below average at the 37th percentile. At this time, his growth demonstrated the 94th percentile for head circumference (50 cm, 19.7 in), the 54th percentile for length (85.7 cm, 33.8 in), and the 100th percentile for weight (15.5 kg, 34.3 lbs).

At 29 months of age, he continued to exhibit depressed speech and language scores, with expressive language scores consistently below receptive language scores across the BSID-IV, PLS-5, and ELM-2. His fine and gross motor skills decreased since he was last seen, with a cumulative motor score at the 14th percentile. During this visit, the mother reported that case 1 had contracted respiratory syncytial virus (RSV) 1 month prior, which developed into pneumonia, but he has since recovered. His head circumference was 51.0 cm (20.1 in) at the 90th percentile. He was 90.2 cm (35.5 in) in length at the 45th percentile and weighed 16.9 kg (37.2 lbs) at the 98th percentile.

### Case 2

#### Diagnosis

Case 2 was diagnosed with 48,XXYY prenatally via amniocentesis. After birth, his diagnosis was confirmed by karyotype. Prenatal ultrasound testing also revealed a Dandy–Walker cyst. He received three neurodevelopmental evaluations at 14, 27, and 39 months of age, a medical genetics and pediatric neurology evaluation, a pediatric speech and language evaluation, and a physical therapy evaluation at 14 months of age. Case 2 received EHT at 14, 15, and 16 months of age but did not receive any intervention services prior to 14 months.

#### Birth history

Case 2 was the product of a 39-week gestation to a 42-year-old, G4P2Mis2, Hispanic woman and her white, nonconsanguineous mate. Due to fertility issues, past miscarriages, and an insufficient number of eggs, conception was completed through egg donation. There is no health history known about the egg donor. Mother noted that fetal movement was felt later than during her first pregnancy and with decreased activity. Mother also reported stress and polyhydramnios during pregnancy. Case 2 was delivered via cesarean section due to complications of the mother’s previous pregnancy, including fever, meconium, and sepsis. He weighed 3.3 kg (7.3 lbs, 46th percentile) and measured 48.3 cm (19.0 in, 20th percentile) in length.

#### Perinatal and early childhood history

This patient was hospitalized in the NICU for 6 weeks following delivery. He was born with a cleft of the soft palate, obstructing his ability to breastfeed, as well as a congenital muscular torticollis on the left side. He exhibited poor feeding habits, a slight cardiac murmur, and transient tachypnea. He was evaluated by a pediatric pulmonologist for his transient tachypnea, which resolved with no complications. The patient further required oxygen via nasal cannula for 3 weeks and received nourishment via a nasogastric (NG) tube. He presented with abnormal posturing, subsequently requiring an EEG, which was normal. At 13 months, pressure equalization (PE) tubes were placed in his ears after the discovery of excessive fluid.

#### Medical genetics/pediatric neurology evaluation— 14 months

Medical history is notable for a febrile seizure at 1 year of age while the family was visiting relatives. He was seen in a local urgent care and released. There was a history of 1 week of fever and upper respiratory symptoms, though no workup was completed. At 13 months of age, he had surgery to repair the cleft palate and a left undescended testicle. Not long after this procedure, he had a mild course of COVID-19, as did the entire family.

On examination at 14 months, he had some mild dysmorphic features, which we have seen in SCA. He was noted to have frontal bossing, slight posterior rotation of the ears, and upslanting palpebral fissures. Hemivertebrae were noted on X-ray. He appeared to have gravitational instability and retracted the upper extremities when the head was moved. He could sit but was unable to get into the sitting position. He did not consistently take weight on his feet and had decreased lateral propping reflexes. He had moderate truncal hypotonia and appeared to have an aversion to certain types of movement with a vestibular component. He had not yet received EHT.

#### Physical therapy evaluation— 14 months

At 14 months, the patient’s mother reports that the patient will play in a sitting position for short periods once placed in the position. The preferred position was supine, where he was able to play with his feet and reach for toys in midline. He showed limited pushing of his feet against the surface while in supine. He rolled to prone with physical cueing. In prone, he needed assistance to attain the on-elbows position, and his hands remained fisted. There was significant head lag in pull-to-sit. Once sitting, he showed a significant C-curve of the spine, with a tendency to push his legs into extension. He tolerated movement into sitting positions with knees flexed as well as assisted 90/90 sitting on a small cushion. There were no independent sitting transitions. He did not attempt to pull to stand, and when placed in the position, he had difficulty maintaining weight on his legs. The ankles showed total medial collapse. He tolerated assisted kneeling against cushions as well as assisted four-point positions to play with toys. He did show beginning balance reactions in sitting once he began to play with toys. He showed gross motor skills between 3 and 5 months, and there was a general paucity of movement and lack of body awareness.

Overall, the patient showed moderate hypotonia and joint hyperextensibility. There is remaining torticollis with limited left cervical rotation to 40°. Trunk extension is weak but improved when he was in supported kneeling and not completely horizontal on the floor. He showed toe clawing in most weight-bearing positions of the feet. The lower extremities were weak, especially in active knee and hip extension. Passive range of motion was normal, with indications of hyperextensibility in the knees, ankles, elbows, and wrists. He showed a tendency toward a resting posture of internal rotation of the lower extremities.

The BSID-IV was completed at 14 months for this patient and indicates standardized motor scores, as shown in **Table 2**.

**Table 2:** Case 2 neurodevelopmental testing.

Bayley Scales of Infant and Toddler Development, 4th Edition (BSID-IV)				
Subtest		14 months	27 months	39 months
Cognitive	Scaled Score	6	1	3
	Composite Score	80	55	65
	**Composite Percentile Rank**	**9**	**0.1**	**1**
Language	Receptive Language Scaled Score	7	2	1
	Expressive Language Scaled Score	3	1	1
	Composite Score	72	51	45
	**Composite Percentile Rank**	**3**	**0.1**	**0.1**
Motor	Fine Motor Scaled Score	1	5	5
	Gross Motor Scaled Score	1	1	1
	Composite Score	60	61	61
	**Composite Percentile Rank**	**0.4**	**0.5**	**0.5**
Preschool Language Scale - 5 (PLS-5)				
Subtest		14 months	27 months	39 months
Auditory Comprehension	Standard Score	96		69
	**Percentile Rank**	**39**		**2**
Expressive Communication	Standard Score	66		60
	**Percentile Rank**	**1**		**1**
Early Language Milestone Scale - 2 (ELM-2)				
Subtest		14 months	27 months	39 months
Expressive Language	Standard Score	69	69	
	**Percentile Rank**	**2**	**2**	
Receptive Language	Standard Score	69	69	
	**Percentile Rank**	**2**	**2**	

Values indicated in bold are percentile rank values based on the standard scoring manuals for each test.

#### Pediatric oral motor/speech and language evaluation— 14 months

In communication, this patient interacted eagerly with his family and the therapist. He made eye contact along with social smiles consistently. He vocalized and moved his body to initiate interaction and gain attention. He sustained appropriate attention, turn-taking, and eye contact during play routines. He approximated blowing kisses with his father. Low muscle tone of the labial, lingual, and buccal musculature was observed. There was limited dissociation of movement in the orofacial complex overall; however, he did demonstrate retraction of the lips to smile. He also demonstrated movement of the upper face when smiling to express joy. Mandibular movement was observed with labial movement at this time. He appeared to combine-breathe (oral and nasal) with lips sealed intermittently when at rest. When actively engaged in interaction, he demonstrated improved lip closure.

Nasal breathing and lip closure are expected for his age, but given the low muscle tone, it is not uncommon to see an open oral resting posture. An intraoral exam revealed excessively thick tissue at the maxillary labial frenum. Examination of the lingual frenulum revealed tight, short tissue that attaches to more than half the underside of his tongue, extending past the mid-tongue and near the tongue tip. These frenal attachments appeared to severely restrict the range of motion in his upper lip and tongue. Mandibular labial and buccal frenula appeared to be within normal limits, with no concerns at this time. Additionally, he demonstrated a high palatal vault, secondary to low-lingual rest posture.

When awake, he exhibited tongue-sucking. His parents shared that the “sound” appeared postoperatively but suspected that the oral habit began earlier and was not audible because of the cleft. Both of these difficulties can be secondary to restriction at the base of the tongue. When asleep, his tongue was observed on the floor of his mouth. With manipulation of the floor of his mouth from under his chin with the mouth closed, elevation was achieved in approximately 80% of the attempts. Minimal lingual–palatal suction was achieved after continued attempts but was difficult to maintain. The restricted tissue appears to keep the base of the tongue from elevating and remaining elevated.

The cleft in his soft palate caused early complications with feeding. In the NICU, he had an NG tube, and his mother exclusively pumped for 2 months. A modified barium swallow study was completed at 5 weeks, and the determination was “an irregular sucking pattern”. Given the lingual restriction observed, that was to be expected because the motility at the base of the tongue is required for rhythmic movements for balanced sucking patterns. He progressed through all nipple levels of the Dr. Browns bottles to the “Y” cut level. He took cereal in his bottle to increase caloric intake but not enough to significantly thicken the liquid. He was appropriately self-regulating with the flow of the bottle. He was not struggling with gastroesophageal reflux disease (GERD) at this time.

#### Neurodevelopmental evaluation—14, 27, and 39 months

Case 2 received three neurodevelopmental evaluations at 14, 27, and 39 months of age. The BSID-IV was completed during all three evaluations. Additionally, the ELM-2 was completed at 14 months, and the PLS-5 was completed at 27 months (**Table 2**).

At 14 months of age, he showed depressed scores in the majority of developmental domains. He had age-appropriate receptive language skills on the PLS-5 (39th percentile) but significantly depressed expressive language skills at the first percentile. His language skills on the ELM-2 and the BSID-IV remained below the third percentile for both receptive and expressive language skills. This speech and language profile puts case 2 at risk for IDD, which is seen in many children with variant forms of X and Y chromosomal disorders (30). This is often a precursor to CAS, which is common in variant forms of SCAs (31). Similarly, he demonstrated cognitive skills at the ninth percentile and motor capabilities at the 0.4th percentile on the BSID-IV. At the time of this evaluation, he weighed 9.8 kg (21.5 lbs, 34th percentile), measured 75.7 cm (29.8 in, 13th percentile) in length, and had a head circumference of 48.3 cm (19.0 in, 89th percentile). He is quite complex in his neurodevelopmental presentation for his young age and had not received EHT or any early intervention services prior to this evaluation.

At 27 months of age, case 2 was seen for a follow-up neurodevelopmental evaluation. He received EHT prior to this evaluation, but early intervention services were extremely limited and inconsistent. Case 2 continued to exhibit depressed scores in all domains of development. He scored below the first percentile in cognition, receptive language, expressive language, fine motor, and gross motor skills on the BSID-IV. On the ELM-2, he scored in the second percentile in both expressive and receptive language. At the time of this evaluation, he weighed 12.4 kg (27.4 lbs, 30th percentile), measured 89.5 cm (35.3 in, 55th percentile) in length, and had a head circumference of 51.0 cm (20.1 in, 92nd percentile).

At 39 months of age, case 2 demonstrated depressed scores in all domains of development; however, he was independently ambulatory and had an additional surgery on his cleft palate, which resulted in fewer ear infections. He also recently began appropriate and intensive intervention services. He scored at or below the first percentile in cognition, receptive language, expressive language, fine motor, and gross motor skills on the BSID-IV. Moreover, on the PLS-5, he exhibited highly depressed skills in receptive and expressive language skills, with scores in the second and first percentiles, respectively.

He showed age-appropriate social skills and repeatedly engaged in social games. He repeatedly attempted to vocalize vowels and consonant–vowel combinations. He continued to have decreased muscle tone in the upper and lower extremities. At the time of this evaluation, he weighed 14.1 kg (31.0 lbs, 32nd percentile), measured 95.3 cm (37.5 in, 32nd percentile) in length, and had a head circumference of 52.0 cm (20.5 in, 93rd percentile).

### Case 3

#### Diagnosis

Case 3 presented with hydrocephalus at approximately 6 months of age. A CMA was ordered in response to the noted developmental delay and loss of overall cerebral white matter volume found on a brain MRI and showed 48,XXYY. He was conceived through IVF, and there were no complications during pregnancy. His mother was taking Lamictal and Synthroid for bipolar I disorder and hypothyroidism during pregnancy.

#### Birth history

He was born to a 30-year-old, G5P2Mis3, white woman and her white, nonconsanguineous mate after 36 weeks of gestation. He had a vaginal, head-first presentation, with APGAR scores of 8 and 8 at 1 and 5 min, respectively. He had a birth weight of 2.7 kg (5.8 lbs, fifth percentile), a length of 48.3 cm (19.0 in, 25th percentile), and a head circumference of 34.0 cm (13.4 in, 50th percentile).

#### Perinatal history

At 10 min of life, he was tachypneic and tachycardic, requiring CPAP 6 and surfactant. During the night, he experienced several apneic episodes that increased in frequency and required intubation. He was extubated after just a few minutes but showed persistent desaturation, requiring prolonged use of a nasal cannula. The origin of this hypoxemia is most likely due to prematurity and mild respiratory distress syndrome prior to surfactant administration. By the time of discharge, he was tolerating room oxygen for more than 48 h without desaturation events. He did not receive EHT.

#### Medical genetics/pediatric neurology evaluation— 1 and 4 months

He was seen by pediatric neurology at 2 days old for apneic episodes and the possibility of seizures. He was found to have a left shift on his complete blood count (CBC), indicating concern for meningitis or new-onset seizure. During apneic episodes, he was noted to posture his upper extremities with his hands fixed, prompting further concern for seizure-like activity. He was transferred to a pediatric tertiary care hospital when he presented with another apneic episode that included “extended arms, with thumbs bent inward, eyes looking upward, and rhythmic tongue movements”. He was placed on Ativan and received a follow-up with neurology.

The neurological evaluation revealed that he was encephalopathic with a symmetric face and truncal hypotonia. He showed minimal grimace to noxious stimuli and moved slightly to touch. He was unable to elicit a sucking reflex due to intubation. He showed 1+ bilateral patellar and biceps reflexes, as well as an upgoing plantar reflex. He presented with a hypotonic, “frog”-leg position and demonstrated withdrawal to noxious stimuli in all four limbs symmetrically. An EEG was recommended and revealed one electrographic seizure early in admission. However, no additional seizure-like activity was detected after this, despite persistent intermittent desaturations. He was weaned off phenobarbital, and no additional seizure activity was reported. An MRI showed mild, nonspecific enlargement of the lateral ventricles.

Case 3 received a follow-up neurology examination at approximately 4 months of age, during which he showed decreased head control for his age. He presented with hydrocephalus, brachycephaly, plagiocephaly, and torticollis. All reflexes were present and appropriate. He had good visual attention, a social smile, and normal movement patterns. He fixated on and followed objects and had easy, sustained eye contact. He received a repeat brain MRI at this time, which revealed decreased cerebral white matter volume compared to the neonatal period. Genetics was consulted about the possibility of a neurodegenerative disorder, and a karyotype and CMA were ordered.

The karyotype revealed the diagnosis of 48,XXYY at approximately 6 months of age. At this time, his neurologic exam reported no dysmorphic features and appropriate social babbling with no consonant sounds. He showed good visual tracking abilities. He was able to bring his hand and thumb to his mouth, place his hands on a bottle at midline while feeding, and had good head control despite the brachiocephalic shape and increased head size. He was not yet reaching into forward flexion and would roll from the stomach to the side unintentionally. He weighed 3.7 kg (8.2 lbs, < 1%), measured 49.4 cm in length (16.9 in, < first percentile), and had a head circumference of 43.0 cm (16.9 in, 70th percentile) during this visit.

Growth measurements for case 3 were taken again at 8 months. He weighed 5.4 kg (11.9 lbs, < first percentile) and had a head circumference of 46.5 cm (18.3 in, 90th percentile).

### Case 4

#### Diagnosis

Case 4 was prenatally screened and revealed to be at high risk for 47,XXY via NIPS, and his mother chose to forego amniocentesis confirmation. After birth, a diagnosis of 48,XXYY was confirmed through karyotype testing. He received three neurodevelopmental evaluations at 5, 7, and 16 months, a pediatric physical therapy evaluation at 6 months, and a pediatric oral motor as well as speech and language evaluation at 7 months. Case 4 received EHT at 5, 6, and 7 months.

#### Birth history

Case 4 was born to a 37-year-old, G7P5Mis2, white woman. The mother reported low weight gain and bleeding throughout her pregnancy. She had high human chorionic gonadotropin (hCG) levels around 6–7 weeks and was subsequently seen in the emergency room. Fetal movement was felt at 24 weeks, and no other pregnancy problems were reported. Mother was induced after over 2 days of labor, and case 4 was born with a head-first vaginal delivery at 39 weeks, weighing 3.15 kg (6.9 lbs, 34th percentile) and measuring 51.0 cm (20.1 in, 72nd percentile) in length.

#### Perinatal history

Mother and infant were hospitalized for 2 days after birth. Case 4’s breathing was monitored while in the hospital because of “a squeaking sound while crying”, according to parental report. He was fed formula for the first 7 days after birth due to trouble latching but was breastfed from then on. At 2 months, he began treatment for GERD. Parents reported a clogged tear duct in his left eye. He was diagnosed with torticollis on the right side and received three dosages of EHT between 4 and 7 months.

#### Physical therapy evaluation— 6 months

The patient played in prone and was able to reach for toys, more so on the left. His hands were fisted periodically, with the left hand open more often. He attempted the extended arm position and was successful if the pelvis was stabilized. He also showed some lateral weight to the left. In supine, he had trouble crossing the midline with the right arm. He was very eager to play with his feet and did a lot of kicking and pushing his feet against surfaces. When placed in a sitting position, he was able to prop sit for several minutes. When placed in a standing position, he took weight briefly, which is normal for his age. The head righting reaction was diminished on the right.

Case 4 showed overall mild hypotonia and joint hyperextensibility. There was significant right torticollis with associated left posterior cranial flattening, elevated and retracted right shoulder, and a smaller right eye orbit. The head is held in right lateral flexion, which is also seen on the right side of his trunk. Passive range of motion was normal with the exception of the cervical spine, and he showed joint hyperextensibility in the knees, ankles, elbows, wrists, and fingers.

The BSID-IV was completed at 6 months for this patient and indicates standardized motor scores, as shown in **Table 3**.

**Table 3:** Case 4 neurodevelopmental testing.

Bayley Scales of Infant and Toddler Development, 4th Edition (BSID-IV)				
Subtest		5 months	7 months	16 months
Cognitive	Scaled Score	7	10	11
	Composite Score	85	100	105
	**Composite Percentile Rank**	**16**	**50**	**63**
Language	Receptive Language Scaled Score	8	7	4
	Expressive Language Scaled Score	10	7	7
	Composite Score	95	83	75
	**Composite Percentile Rank**	**37**	**13**	**5**
Motor	Fine Motor Scaled Score	11	6	8
	Gross Motor Scaled Score	11	4	9
	Composite Score	106	72	91
	**Composite Percentile Rank**	**66**	**3**	**27**
Preschool Language Scale - 5 (PLS-5)				
Subtest		5 months	7 months	16 months
Auditory Comprehension	Standard Score	96	91	96
	**Percentile Rank**	**39**	**27**	**37**
Expressive Communication	Standard Score	102	66	101
	**Percentile Rank**	**55**	**1**	**53**
Early Language Milestone Scale - 2 (ELM-2)				
Subtest		5 months	7 months	16 months
Expressive Language	Standard Score	102	69	98
	**Percentile Rank**	**55**	**2**	**45**
Receptive Language	Standard Score	125	110	75
	**Percentile Rank**	**95**	**75**	**5**

Values indicated in bold are percentile rank values based on the standard scoring manuals for each test.

#### Pediatric oral motor/speech and language evaluation— 7 months

At the time of the speech and language evaluation, case 4 was being breastfed on demand. By parent report, he nurses frequently for short sessions and latches on and off constantly. When using a bottle, he has difficulty with a lip seal. Purees were introduced into his diet at 4.5 months, but he struggles with eating from a spoon and typically pushes and spits the food out. Parents reported difficulties in the following areas while breastfeeding: shallow latch, consistent reflux or spitting up, cluster feeding, preference for side of position, falling asleep while feeding, and choking/coughing. Parents reported difficulties in the following areas while bottle feeding: shallow latch, consistent reflux or spitting up, milk dribbling out of the mouth, and choking/coughing.

Case 4 signaled that he was hungry by vocalizing and rooting. He latched to the breast quickly with a shallow latch. No nasal congestion was observed today. His breathing was quiet, with no signs that it was labored. He frequently popped off the nipple purposefully but, at times, would slide off and lose the suction of his latch. In both scenarios, he appeared fatigued. This behavior is common in babies with oral weakness.

Observations of the orofacial complex were made throughout the session. Low tone of labial, lingual, and buccal musculature was observed. There was limited dissociation of movement in the orofacial complex overall; however, he demonstrated retraction of his lips to smile. He also demonstrated movement of the upper face when smiling to express joy.

Asymmetry of the face was observed and appeared positional as well as structural. His jaw opened symmetrically. The emergence of tongue lateralization with delayed reflex when elicited was observed. A phasic bite is easily elicited with munching-chew emerging. A combination of mouth and nasal breathing was observed. Nasal breathing and lip closure are expected for his age, but given the low muscle tone, it is not uncommon to see an open oral resting posture. His tongue is in the low-forward resting position. When the range of motion was assessed, the tongue was difficult to lift off the floor of the mouth. The frenulum was tight and short, with tension at the posterior portion being most significant. Blanching at the lingual frenulum was seen immediately with minimal elevation of the tongue. As expected, his palate was high and narrow. Case 4’s oral resting posture is detrimental to his orofacial development.

His maxillary frenal were also assessed. When assessed with the upper lip down (at rest), tension was felt at both the right and left maxillary buccal and labial frenula. His upper lip was difficult to lift and guide toward the nose in order to view the maxillary frenal. Blanching and a limited range of motion at the maxillary lip and buccal frenal were observed. The tension in the upper lip makes it easier for him to retract it for smiling.

These frenal attachments appear to severely restrict the range of motion in his upper lip and tongue. They make feeding and speech development more difficult because they do not allow the lingual, labial, and buccal muscles the movement needed to develop strength, coordination, and dissociation from the jaw, which is necessary for articulation and feeding.

#### Neurodevelopmental evaluation—5, 7, and 16 months

Case 4 received three evaluations within the first 2 years of life at 4, 7, and 16 months. The following neurocognitive assessments were completed during each evaluation: the BSID-IV, the PLS-5, and the ELM-2 (**Table 3**).

At 5 months, he presented with speech and language skills within normal limits on the ELM-2, PLS-5, and BSID-IV. He presented with age-appropriate cognitive skills on the BSID-IV. He exhibited decreased muscle tone in the upper extremities, lower extremities, and core. Both fine and gross motor scores on the BSID-IV were within normal limits. All primitive neonatal reflexes were within normal limits, except for some restriction in the asymmetric tonic neck reflex due to the torticollis. He showed age-appropriate social, attentional, and behavioral skills. His head circumference was 39.5 cm (15.6 in, < first percentile). He was 64.1 cm (25.3 in) in length at the 23rd percentile and weighed 6.9 kg (15.3 lbs) at the 25th percentile.

At 7 months of age, he was seen again and had received EHT since his last visit. Parents report he was still having difficulty with his left tear duct. He had three ear infections in the past month and experienced rashes secondary to a yeast infection from the antibiotics. Scores from the PLS-5 and ELM-2 indicate that he is within normal limits for receptive language but delayed in expressive language, at the first and second percentiles. Scores from the BSID-IV show he is below average in both receptive and expressive language, with a more significant delay in expressive language. These scores are consistent with an evolving Childhood Apraxia of Speech (CAS) preceded by an IDD, which is common in children with SCAs. His cognitive and fine motor skills remain age-appropriate according to the BSID-IV, but his gross motor skills are below average. His torticollis shows improvement with less muscle tightness and more mobility; however, some asymmetry is still noted. When placed in standing, his toes pull into an extensor posture positioning, which is atypical and not common in infants with 48,XXYY. His head circumference was 41.0 cm (16.1 in, < first percentile). He was 71.1 cm (28.0 in) in length at the 65th percentile and weighed 8.3 kg (18.3 lbs) at the 39th percentile.

At 16 months of age, case 4 was seen for another follow-up neurodevelopmental evaluation. His PLS-5 scores show that he is within normal limits for both expressive and receptive language, with receptive language slightly lower at the 37th percentile. Scores from the ELM-2 indicate that he is within normal limits for expressive language but delayed in receptive language at the fifth percentile. Scores from the BSID-IV show that he is below average in both receptive and expressive language, with a more significant delay in receptive language. Receptive language scores may have been lower due to compliance and attention issues. His cognitive, fine motor, and gross motor skills remain age-appropriate according to the BSID-IV. His head circumference was 44.5 cm (17.5 in) at the second percentile. He was 80.0 cm (31.5 in) in length at the 34th percentile and weighed 9.8 kg (21.6 lbs) at the 22nd percentile.

### Case 5

#### Diagnosis

Case 5 was diagnosed postnatally via whole exome sequencing and karyotype testing at 34 months of age. He received one neurodevelopmental evaluation at 35 months, a pediatric physical therapy evaluation at 36 months, and a pediatric oral motor as well as speech and language evaluation at 36 months. All scores calculated and observations made during the evaluations were based on developmental age. Case 5 received EHT at 38, 39, and 40 months.

#### Birth history

Case 5 was born to a 28-year-old, G2P2Mis0, white woman. No pregnancy problems were reported until the mother’s membranes prematurely ruptured at 34 weeks. Labor was induced, leading to a head-first vaginal delivery. At birth, case 5 weighed 2.5 kg (5.5 lbs, third percentile), measured 43.2 cm in length (17.0 in, first percentile), and had a head circumference of 33.5 cm (13.2 in, 22nd percentile).

#### Perinatal and early childhood history

Case 5 spent 10 days in the NICU after birth due to respiratory distress and clinical sepsis. He was treated and sent home with an apnea monitor for 4 months due to bradycardia. He did not have trouble breastfeeding.

He experienced repeated middle ear infections during his first year, leading to the insertion of PE tubes at 8 months of age. At 25 months, case 5 underwent an MRI, which revealed macrocephaly, chronic microhemorrhages, and mild lateral ventriculomegaly. He was seen by an ophthalmologist due to esotropia, which has since been resolved. Parents report a diagnosis of sleep apnea as well. He had an orchidopexy procedure at 32 months and was hospitalized due to RSV at 32 and 34 months. He currently uses an inhaler as needed.

#### Physical therapy evaluation— 36 months

Case 5 showed hypotonia and joint laxity throughout the evaluation. His active range of motion was normal, but his passive range of motion showed hypermobility, particularly in his hands, wrists, elbows, and knees. The postural screening showed that case 5 had mild right torticollis with the right shoulder slightly elevated.

In supine on the floor, the right leg appears slightly longer, with the left hip mildly hiked. He prefers the W-sit position while in floor play but can easily move into other positions. His standing posture shows a mild rounding of the shoulders with increased lordosis and slight knee recurvatum. Also, while standing, his ankles show mild medial collapse with increased calcaneal eversion and mild toe clawing. These issues are not uncommon in a 3-year-old child with hypotonia and joint hypermobility but should be monitored as he matures. Weight-bearing on his arms shows the elbow and wrist hypermobility as well as some finger clawing. There is increased tibial torsion in the lower extremities. He also shows a mild asymmetry in the legs, with the left being slightly weaker than the right.

Case 5 shows age-appropriate movement transitions from the floor to standing. He also has a nice squat position for play. While walking and running, he shows increased internal rotation. He initially had trouble walking up and down the stairs, but with time, he improved. He also had trouble stepping over obstacles during some of the games, and this too improved with time; he fell at least three times but resumed the game. He had no problems taking weight on his arms when playing with a large ball.

Case 5’s mother reports that he does not like to be upside down. As he did the obstacle course, he had to feel his way through the changes, sometimes tripping over things before he knew where they were. He had trouble catching balls and did not like balls coming toward him; visual tracking of the ball was hard for him. He shows visual–spatial issues and possibly vestibular issues.

#### Pediatric oral motor/speech and language evaluation— 35 months

Case 5 had an NG tube while in the NICU and was later successful with breastfeeding and bottle feeding. He had difficulty transitioning to solid foods. Mom reported that he would occasionally gag, choke, and spit up food. He was successful with purees. At the time of this evaluation, case 5 eats a wide variety of foods with minimal encouragement. He has difficulty with meats and will pull them out of his mouth when he struggles to chew them. He shows the following signs of oral dysfunction: overstuffing food in the oral cavity, pocketing food in the oral cavity, messiness, and spitting food out. These behaviors are typically seen when it is difficult for the child to dissociate movements, which is necessary for bolus formation.

It is clear that he wants to interact with his family members but struggles to make himself understood. He demonstrates understanding of what he was asked during today’s session. He also attempts to communicate verbally. His attempts to communicate sound similar and lack variation in sounds. He lacks articulatory placement and contact, secondary to oral weakness and dysfunction.

Case 5 exhibited an open mouth with a low, forward, lingual oral resting posture. He has a lower tone of the labial, lingual, and buccal musculature than expected for his age. Dissociation of movement during speaking and eating was limited. He has a class III malocclusion (underbite). This bite pattern is common in children with low-lingual rest posture. It is detrimental to remediate his resting posture for orofacial growth and development of articulation and feeding skills. Children with low-lingual resting posture typically have orthodontic relapse because their tongue does not hold the realignment appropriately.

Upon examination of the oral frenal, case 5 demonstrated tethering at the tongue, upper and lower lip, as well as excessive tissue at the buccal connections. Continued diagnostic treatment will be needed to determine which tethered oral tissues are recommended for release. It is suspected that these restrictions are at least part of the cause of a lack of muscle development and dissociation of movement. Restriction of the oral frenal could be preventing the lingual, labial, and buccal muscles from moving as needed to develop strength, coordination, and dissociation from the jaw, which is required to complete the necessary movements for articulation and feeding. If not addressed with functional and possibly structural interventions, compensatory movements will continue to develop, and oral dysfunction will remain a challenge.

He demonstrated mouth breathing, and his mother reports a history of sleep apnea in infancy, which has been resolved; however, he still shows signs of airway difficulty. She shared that, in his sleep, he demonstrates restlessness as well as oral habits associated with airway obstruction (pacifier use at night).

#### Neurodevelopmental evaluation— 35 months

Case 5 received one neurodevelopmental evaluation at 35 months of age. The following neurocognitive assessments were completed during the evaluation: the BSID-IV, the PLS-5, and the ELM-2 (**Table 4**). At the time of evaluation, he had not received EHT.

**Table 4:** Case 5 neurodevelopmental testing.

Bayley Scales of Infant and Toddler Development, 4th Edition (BSID-IV)		
Subtest		35 months
Cognitive	Scaled Score	5
	Composite Cognitive Score	75
	**Composite Percentile Rank**	**5**
Language	Receptive Language Scaled Score	6
	Expressive Language Scaled Score	5
	Composite Score	75
	**Composite Percentile Rank**	**5**
Motor	Fine Motor Scaled Score	8
	Gross Motor Scaled Score	3
	Composite Score	74
	**Composite Percentile Rank**	**4**
Preschool Language Scale - 5 (PLS-5)		
Subtest		35 months
Auditory Comprehension	Standard Score	90
	**Percentile Rank**	**25**
Expressive Communication	Standard Score	92
	**Percentile Rank**	**30**
Early Language Milestone Scale - 2 (ELM-2)		
Subtest		35 months
Expressive Language	Standard Score	69
	**Percentile Rank**	**2**
Receptive Language	Standard Score	69
	**Percentile Rank**	**2**

Values indicated in bold are percentile rank values based on the standard scoring manuals for each test.

Case 5 showed depressed scores on all domains of the three tests. On the BSID-IV, he scored in the fourth percentile for fine and gross motor and the fifth percentile for receptive language, expressive language, and cognition. On the PLS-5, he scored in the 25th and 30th percentiles for receptive and expressive language, respectively. On the ELM-2, he scored in the second percentile for both receptive and expressive language skills. These delays in speech and language acquisition are consistent with his history of PE tubes and chronic ear infections. No signs of ASD were noted during the evaluation. His head circumference was 53.0 cm (20.9 in), at the 99th percentile. He was 91.4 cm (36.0 in) in length at the 21st percentile and weighed 14.9 kg (32.8 lbs) at the 65th percentile.

## Discussion

The case series describes the neurodevelopmental profile of five patients with 48,XXYY, which has not been previously reported in the research literature to our knowledge. These five profiles reveal the wide range of phenotypic presentations in this rarely diagnosed disorder. These findings vary from relatively uncomplicated to complex, contributing novel information on the phenotype of patients with 48,XXYY and highlighting common manifestations seen from infancy to early childhood (**Table 5**). Furthermore, these cases may highlight the intriguing and potentially positive effects of EHT on the neurodevelopmental outcome of patients with 48,XXYY.

**Table 5:** Common manifestations in 48,XXYY, within the first 39 months of life.

Common features	Present in case 1	Present in case 2	Present in case 3	Present in case 4	Present in case 5
Hypotonia	✔	✔	✔	✔	✔
Joint laxity	✔	✔	X	✔	✔
Torticollis	✔	✔	✔	✔	✔
Seizures	X	✔	✔	X	X
Feeding/oral motor deficits	✔	✔	✔	✔	✔
Tethered oral tissue	✔	X	X	X	X
Frenal attachments	X	✔	X	✔	✔
Sucking/swallowing dysfunction	✔	✔	✔	✔	✔
Pulmonary issues	✔	✔	✔	✔	✔
Respiratory distress at birth	X	✔	✔	X	✔
ASD symptoms	X	X	X	X	X
Speech/language dysfunction	✔	✔	X	✔	✔
Neuromotor dysfunction	✔	✔	✔	X	✔
Large head circumference (> 70th percentile)	✔	✔	✔	X	✔
Maternal miscarriage history	✔	✔	✔	✔	X

Previous research has noted motor dysfunction, including poor coordination and motor planning, in children with 48,XXYY (5, 10). In association with these neuromotor features, truncal hypotonia has been commonly reported in the literature and was observed in four out of five of our cases (2, 3, 5, 32). Our observations also noted joint laxity in four of the cases. Gross and fine motor standardized testing revealed that cases 2, 3, and 5 demonstrated significantly delayed motor milestones, whereas cases 1 and 4 had much milder delays detected early in life. Additionally, all five patients described in this series presented with a torticollis identified early in life, and three of the five patients presented on the right side. The hypotonia and torticollis presentation documented in this case series has not been reported in 48,XXYY but has been previously described in 47,XXY and 49,XXXXY patients (33). Motor development builds the infrastructure for all learning in children, and therefore early treatment of motor delays in infants with 48,XXXY may provide additional support for optimal neurodevelopment (5, 10, 33). Future research is warranted regarding the specific motor dysfunction in 48,XXYY in order to better understand the neuromotor trajectory of this disorder and the impact on learning.

Four of the patients exhibited head circumference measurements that were consistently above the 70th percentile between 0 and 35 months (**Table 6**). Case 4 showed head circumference measurements below the third percentile with a diminished paternal head circumference. The impact of familial microcephaly is the likely contributory factor to the diminished head circumference in case 4. Our findings are contrary to the Tartaglia et al. (2) study, which noted that participants with 48,XXYY below 10 years old showed a head circumference mean below the 50th percentile, while age groups 11–20 years old and older were above the 50th percentile. These findings, coupled with our observations of consistently increased head circumference in four of our patients, warrant further investigation into the impact of accelerated head circumference on learning and neurodevelopment.

**Table 6:** Height (HT), weight (WT), and head circumference (HC) measurements of cases 1–5.

Patient	Age (months)	HC (cm)	HC (in)	HC percentile	HT (cm)	HT (in)	HT percentile	WT (kg)	WT (lbs)	WT percentile
Case 1	Birth				49.5	19.5	42	3.1	6.8	30
	2.0	40	15.7	88	21.3	8.4	4	4.6	10.0	15
	3.0	41.2	16.2	73	59.4	23.4	19	5.9	13.0	27
	8.9	46.0	18.1	79	70.5	27.8	27	9.9	21.8	84
	12.8	47.5	18.7	82	76.2	30.0	41	11.5	25.4	93
	21.3	50.0	19.7	94	85.7	33.8	54	15.5	34.3	100
	29.5	51.0	20.1	90	90.2	35.5	45	16.9	37.2	98
Case 2	Birth				48.3	19.0	20	3.3	7.3	46
	14.5	48.3	19.0	89	75.7	29.8	13	9.8	21.5	34
	27.4	51.0	20.1	92	89.5	35.3	55	12.4	27.4	30
	39.0	52.0	20.5	93	95.3	37.5	32	14.1	31.0	32
Case 3	Birth	34.0	13.4	50	48.3	19.0	25	2.7	5.8	5
	4.7	43.0	16.9	70	49.4	16.9	0	3.7	8.2	0
	7.4	46.5	18.3	90				5.4	11.9	0
Case 4	Birth				51.0	20.1	72	3.1	6.9	34
	4.9	39.5	15.6	0.8	64.1	25.3	23	6.9	15.3	25
	7.8	41.0	16.1	0.4	71.1	28.0	65	8.3	18.3	39
	16.9	44.5	17.5	2	80	31.5	34	9.8	21.6	22
Case 5	Birth	33.5	13.2	22	43.2	17.0	1	2.5	5.5	3
	35.4	53.0	20.9	99	91.4	36.0	21	14.9	32.8	65

There have been various reports associating 48,XXYY with an increased risk for ASD (9, 14, 17). A literature review of 95 participants with 48,XXYY reported that 23% of these individuals had a previous diagnosis of ASD (14). However, the generational family pedigrees were not reported in the review; therefore, it is difficult to discern whether the incidence of ASD is secondary to the presence of ASD in the family. ASD is a highly heritable disorder, with studies calculating a heritability rate between 64% and 91% (34, 35). Our five cases were each evaluated and monitored for any early signs of ASD via the Comprehensive Autism Spectrum Screening for Infants (CASS-i), as well as their pediatric neurological evaluations in conjunction with the DSM-5-T (36). Based on the pediatric neurological evaluations, none of the five patients showed early signs of ASD. The CASS-i describes four behaviors indicating an at-risk status for ASD at 9 months: atypical head–neck righting reflex, turn to the sound of their name, head lag on pull to sit, and acceleration of head circumference between 3 and 9 months above the 75th percentile (37). While there were head circumference measurements in cases 1, 2, 3, and 5 above the 75th percentile, acceleration of head circumference was not present in any of the cases. Based on the neurological evaluations and the results of the CASS-i, these infants appear to be low risk for ASD. Therefore, we believe that the increased head circumference of each child is linked exclusively to their 48,XXYY karyotype.

Furthermore, case 2 did show significant head lag in pull to sit and a sluggish head-tilt reflex, indicating that he is at a slightly increased risk based on two of the four biomarkers on the CASS-i. It is possible that these observations are an early indication of ASD; however, they may be related to his hypotonia and lack of EHT, as his social skills and interactions were appropriate for his age. Therefore, all five cases presently appear at low risk for ASD but are being followed by the multidisciplinary team of specialists to assess this risk over time. Since three of the five cases were identified prenatally, it is also possible that they have a milder presentation than those identified postnatally, which is often seen in 47,XXY (5, 21).

Feeding and oral motor deficits were present in all five cases. Sucking/swallowing dysfunction is most evident in children who lack lingual–palatal contact secondary to decreased oral motor muscle tonus, which we observed in four out of five patients in this series. The tethered oral tissues in case 1 and the frenal attachments in cases 2, 4, and 5 severely restricted the range of motion of their upper lip and tongue. These complications compromise feeding and speech development by restricting movement of the lingual, labial, and buccal muscles if untreated (38, 39). These muscle movements are necessary to develop strength, coordination, and dissociation from the jaw needed to facilitate satisfactory oral motor movements for articulation and feeding (38–40). Case 3 exhibited a sucking/swallowing dysfunction. He did not receive an oral motor evaluation, so the tone of the oral musculature was not noted; however, decreased muscle tonus in the oral motor area is highly suspected because of the sucking/swallowing dysfunction. The presence of these complications in all five cases warrants further research but does demonstrate the need for early and comprehensive feeding and oral motor evaluations in infants with 48,XXYY. These early feeding issues increase vulnerability for later speech delays and may be contributory toward the early indications of IDD and later presentations of CAS, which can contribute to later behavioral dysfunction commonly associated with this disorder. Further investigation is warranted into the myofunctional aspects of feeding and speech development and its impact on later behavioral manifestations.

All five patients exhibited pulmonary issues in their first two years of life. Tartaglia et al. (2) found that 46% of the 48,XXYY males in their study were hospitalized for respiratory infections or asthma, and 16.1% had recurrent hospitalizations for respiratory complications. Cases 2, 3, and 5 experienced respiratory distress at birth, while the other two patients experienced respiratory issues later in their first two years. Increased and recurrent hospitalizations for upper respiratory infections have been noted in boys with 47,XXY and 49,XXXXY. This suggests that these airway and respiratory issues may be related to the presence of an additive X, although the pathophysiology of this dysfunction is not well understood at this time.

Another trend seen throughout this case series is a history of miscarriage. In four out of five cases, mothers reported having two or more miscarriages prior to birthing their son with 48,XXYY. The relationship between miscarriage history and birthing a son with 48,XXYY is under-investigated in the current literature. However, in Moser et al. (41), researchers explored the incidence of miscarriage in mothers of children with variants of Klinefelter syndrome (47,XXY, 48,XXXY, and 49,XXXXY) and found that there is an increased incidence within and across variant groups. This study showed an increased incidence of miscarriage in mothers of children with SCAs, which is consistent with the findings in this study.

Four out of five patients in this series received EHT during infancy or early childhood. No definitive conclusion can be drawn as to whether this treatment had an effect on the neurodevelopmental outcome of each patient yet, because of the young age of this group. It is of note that case 1 exhibited the best neurodevelopmental outcome; he was prenatally diagnosed, received EHT at the optimal time, was enrolled in early intervention services quite early, and had few medical complications. Previous research studies have hypothesized that numerous factors influence the neurodevelopmental outcomes of males with additive Xs, and this case series provides further support for this hypothesis. The cases in this series show motor, speech, and language impairments similar to other KS variants. There is limited literature describing the phenotype of boys with 48,XXXY due to its rarity; however, these patients appear to have more complex behavioral issues than their counterparts with 47,XXY.

This series highlights novel findings in the phenotypic profile of the infant with 48,XXXY, as well as findings that are common to the older child with 48,XXYY. Our study further describes the early neurodevelopmental profile of a rare SCA and supports the need for comprehensive and multidisciplinary assessment of these infants. Future research is warranted to explore the potential association between neurodevelopment and EHT in patients with 48,XXYY. Additional research should also focus on determining the optimal timing of treatment, as well as the continued effect of testosterone treatment on children with 48,XXYY into adolescence and adulthood.

## Data Availability

The raw data supporting the conclusions of this article will be made available by the authors, without undue reservation.
